# Aldo Molinari: the wedding of the giant Almiro Crema in Torino

**DOI:** 10.1007/s40618-021-01644-y

**Published:** 2021-07-21

**Authors:** W. W. de Herder

**Affiliations:** Department of Internal Medicine, Sector of Endocrinology, Rg520, Erasmus MC, Dr. Molewaterplein 40, 3015 GD Rotterdam, The Netherlands

**Fig. 1:**
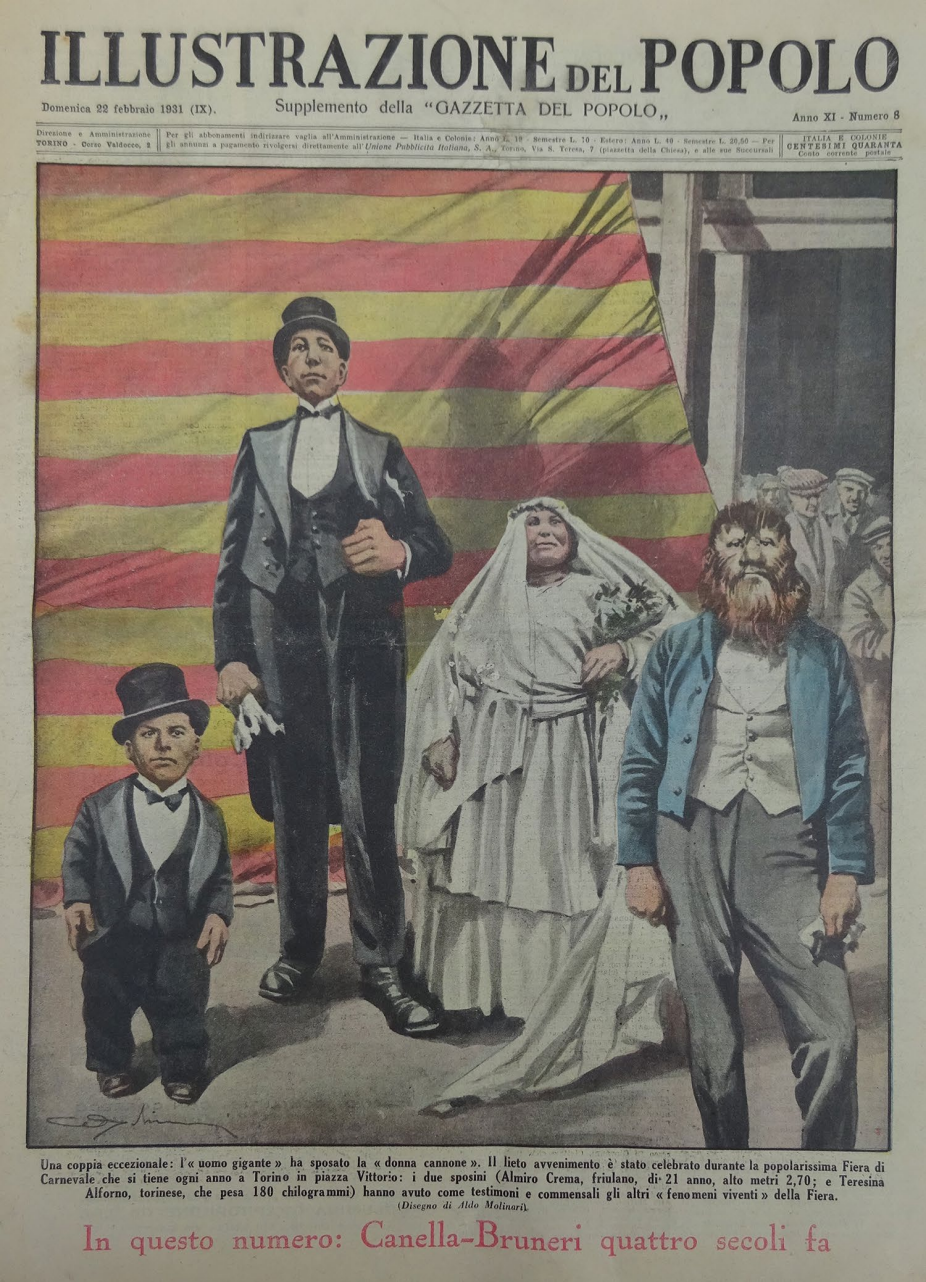
Frontcover of “Illustrazione del Popolo”, 22 February 1931

Front cover drawing by Aldo Molinari (1885–1959) of “Illustrazione del Popolo” dating 22 February 1931, depicting the wedding party of Almiro Crema and Teresina Alfonso (Fig. 1).

**Fig. 2:**
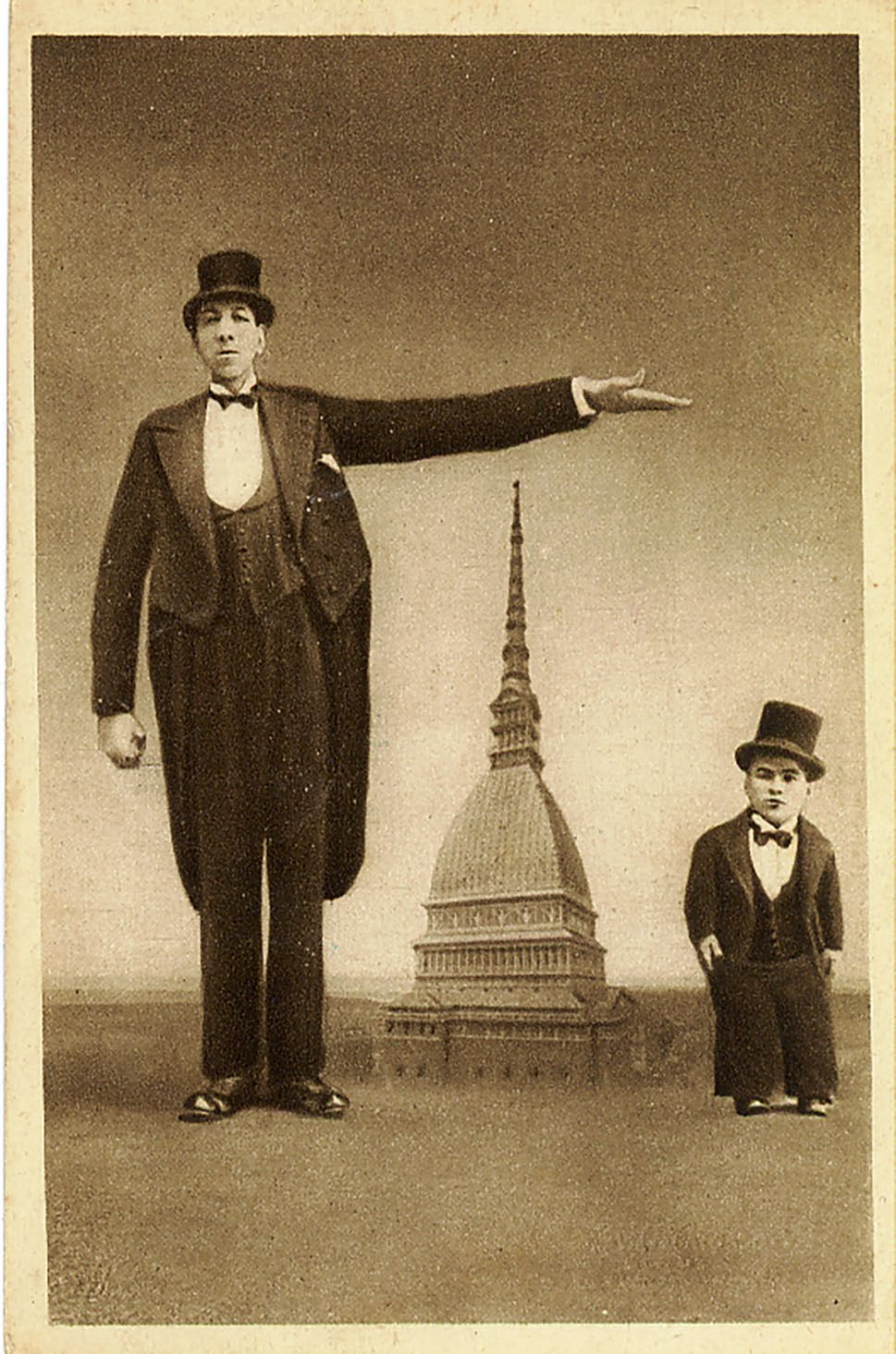
Almiro Crema and “Mister Totò” in front of the “Mole Antonelliana” (Torino)

**Fig. 3:**
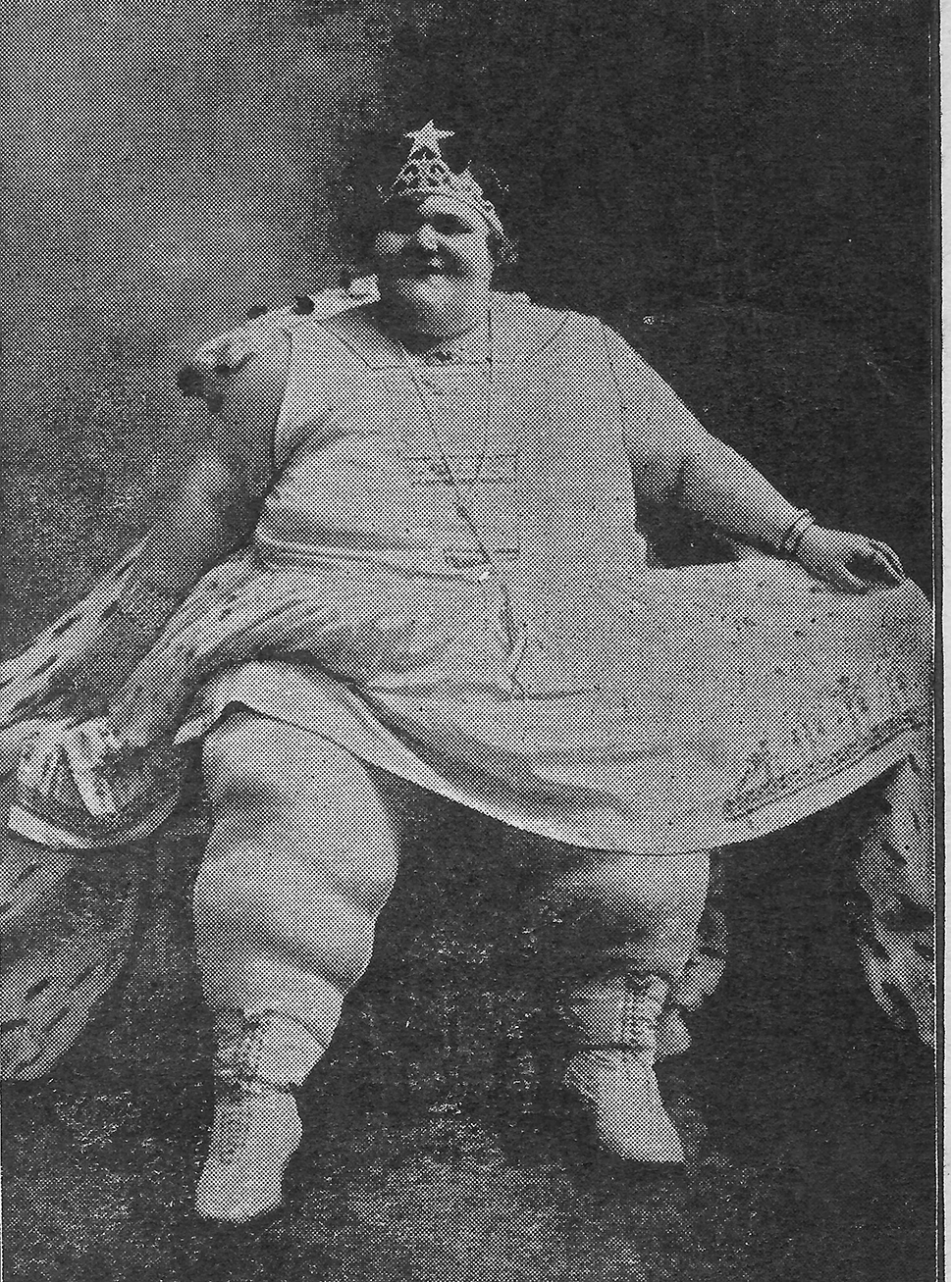
Térésina Alfonso

**Fig. 4:**
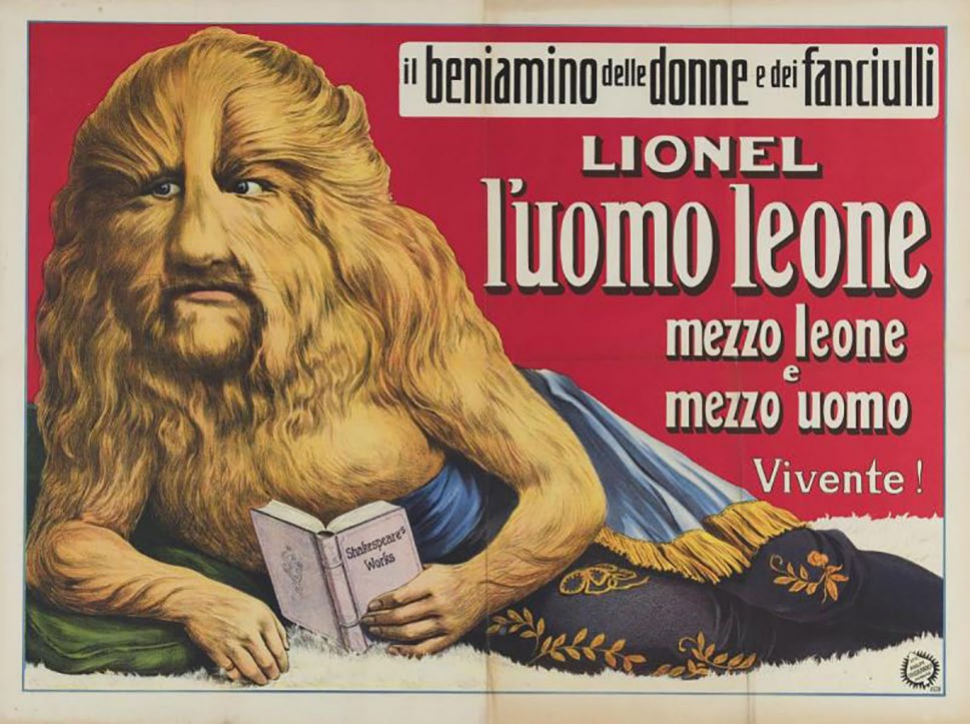
Italian advertisement for Stephan Bibrowski = “Lionel the Lion-faced Man”

Almiro Crema was an acromegalic giant born in 1900 (but some information claims his birth year was 1910). His final height was claimed to be 2.70 m., but his real height must have been around 2.40 m., or less. He died of pneumonia in 1944 and his bones were buried in Casale di Scodosia (Padova, It). During life, he was known as Gigante Golia. He received the “medaglio d’oro—gran distintivo” as “l’homo piu grande da mondo” because of his great height (Fig. 2). He worked in the circus together with “Mister Totò”, who was only 68 cm. tall (Fig. 2). In the circus, he also met his future wife Térésina Alfonso who was morbidly obese and weighed more than 200 kg (Fig. 3). They married in 1931 in Torino (Fig. 1). The front-page picture also shows at the right a male with most probably congenital generalized hypertrichosis (Fig. 1). The Polish-born Stephan Bibrowski (1890–1932), better known as “Lionel the Lion-faced Man”, was also a famous sideshow and circus performer who also performed in Italy in those days (Fig. 4).

Some of the relics of Almiro Crema can be found in the museum “Villa Correr” in Casale di Scodosia (https://it.wikipedia.org/wiki/Villa_Correr) (Figs. 1, 2, 3, 4).

All pictures are from the collection of W.W. de Herder.

